# Efficacy of Adolescent Suicide Prevention E-Learning Modules for Gatekeepers: A Randomized Controlled Trial

**DOI:** 10.2196/mental.4614

**Published:** 2016-01-29

**Authors:** Rezvan Ghoncheh, Madelyn S Gould, Jos WR Twisk, Ad JFM Kerkhof, Hans M Koot

**Affiliations:** ^1^ Department of Clinical Developmental Psychology Faculty of Behavioural and Movement Sciences VU University Amsterdam Amsterdam Netherlands; ^2^ EMGO+ Institute for Health and Care Research Amsterdam Netherlands; ^3^ College of Physicians and Surgeons Department of Psychiatry Columbia University New York, NY United States; ^4^ New York State Psychiatric Institute New York, NY United States; ^5^ VU University Medical Center Department of Epidemiology and Biostatistics Amsterdam Netherlands; ^6^ Department of Clinical Psychology Faculty of Behavioural and Movement Sciences VU University Amsterdam Amsterdam Netherlands

**Keywords:** Adolescent, E-learning, Gatekeepers, Learning, Modules, Online Systems, Suicide, Prevention, Training, Web-based, Referral and Consultation

## Abstract

**Background:**

Face-to-face gatekeeper training can be an effective strategy in the enhancement of gatekeepers’ knowledge and self-efficacy in adolescent suicide prevention. However, barriers related to access (eg, time, resources) may hamper participation in face-to-face training sessions. The transition to a Web-based setting could address obstacles associated with face-to-face gatekeeper training. Although Web-based suicide prevention training targeting adolescents exists, so far no randomized controlled trials (RCTs) have been conducted to investigate their efficacy.

**Objective:**

This RCT study investigated the efficacy of a Web-based adolescent suicide prevention program entitled *Mental Health Online,* which aimed to improve the knowledge and self-confidence of gatekeepers working with adolescents (12-20 years old). The program consisted of 8 short e-learning modules each capturing an important aspect of the process of early recognition, guidance, and referral of suicidal adolescents, alongside additional information on the topic of (adolescent) suicide prevention.

**Methods:**

A total of 190 gatekeepers (ages 21 to 62 years) participated in this study and were randomized to either the experimental group or waitlist control group. The intervention was not masked. Participants from both groups completed 3 Web-based assessments (pretest, posttest, and 3-month follow-up). The outcome measures of this study were actual knowledge, and participants’ ratings of perceived knowledge and perceived self-confidence using questionnaires developed specifically for this study.

**Results:**

The actual knowledge, perceived knowledge, and perceived self-confidence of gatekeepers in the experimental group improved significantly compared to those in the waitlist control group at posttest, and the effects remained significant at 3-month follow-up. The overall effect sizes were 0.76, 1.20, and 1.02, respectively, across assessments.

**Conclusions:**

The findings of this study indicate that Web-based suicide prevention e-learning modules can be an effective educational method to enhance knowledge and self-confidence of gatekeepers with regard to adolescent suicide prevention. Gatekeepers with limited time and resources can benefit from the accessibility, simplicity, and flexibility of Web-based training.

**Trial Registration:**

Netherlands Trial Register NTR3625; http://www.trialregister.nl/trialreg/admin/rctview.asp?TC=3625 (Archived by WebCite at http://www.webcitation.org/6eHvyRh6M)

## Introduction

According to the World Health Organization (WHO), approximately 1 million people die worldwide every year due to suicide [1]. Suicide is the second leading cause of death among 10- to 24-year-olds, and according to a WHO report, suicide rates are rising faster among adolescents compared to any other age category [1]. Moreover, for every adolescent suicide, there are at least 40 non-fatal suicide attempts [2]. Thus, the development and deployment of adolescent suicide prevention strategies are crucial.

Recently, it has become widely accepted that gatekeepers can play an essential role in suicide prevention; as a result, the training of gatekeepers has been identified as an important and promising prevention strategy [3-7]. Gatekeepers are professionals who, due to their profession, come in contact with people at-risk for suicide. Thus, the main purpose of training gatekeepers is to educate them in the necessary steps concerning recognition, guidance, and referral of these individuals [5,6,8]. For instance, primary health care providers, school staff, and police are all gatekeepers [3,7]. Several suicide prevention gatekeeper programs are available, which have been widely adopted (eg, Question, Persuade, Refer (QPR); Sources of Strength (SOS); Applied Suicide Intervention Skills Training (ASIST); Yellow Ribbon and safeTALK) [9]. QPR Gatekeeper Training [10] is one of the most well-known and used gatekeeper training programs in suicide prevention [11].

QPR Gatekeeper Training is based on the QPR model, which was developed and introduced in 1995 [10]. According to this model, three simple steps can be employed to prevent suicide attempts. First, gatekeepers must learn to recognize warning signs associated with suicide and learn how to ask questions about the presence of suicidal thoughts and feelings (Question). The earlier that warning signs are recognized and help is received by the at-risk individual, the better the outcome will be. Second, questioning those at-risk for suicide could lead to conversations during which the acceptance of referrals for help can be encouraged (Persuade). Lastly, referrals will lead to early intervention and treatment, which will lead to better outcomes (Refer) [10].

In recent years, several studies have investigated the efficacy of QPR Gatekeeper Training and the results are promising. These studies have targeted various types of gatekeepers, including Veterans Affairs staff, Veterans Health Administration staff, college residence advisers, university faculty staff, and social work students, and have shown that gatekeepers’ actual or perceived knowledge and perceived self-efficacy with regard to suicide prevention improve after attending training [12-18]. Additionally, several research teams have studied the efficacy of QPR Gatekeeper Training in gatekeepers working with adolescents [11,19-21]. A randomized controlled trial (RCT) with an average 1-year follow-up period tested the impact of the training on school staff (health and social services staff, administrators, teachers, and support staff) and showed enhancement of perceived knowledge, perceived efficacy, and preparedness of the trained gatekeepers to perform suicide prevention activities [19]. Another study using a nonequivalent control group design with a 3-month follow-up, demonstrated increased knowledge among trained teachers and counselors working in elementary, middle, and high schools [11]. Another nonequivalent control group design with a 3-month follow-up showed gains in knowledge and self-efficacy among trained school personnel at posttest [20]. Moreover, the self-efficacy gain was maintained at follow-up; however, this was not the case for knowledge. According to the authors, this could be explained by the limited subsample that completed the follow-up measures [20]. Finally, a study targeting faculty and staff who worked regularly with middle and high school students showed that the knowledge of participants increased after completing training [21].

The results of the discussed papers demonstrate that face-to-face gatekeeper training can be an effective strategy in the enhancement of professionals’ knowledge and self-efficacy in adolescent suicide prevention. However, for several reasons, gatekeepers may be prevented from attending training sessions. The most critical barrier for gatekeepers is lack of time and resources to attend face-to-face training sessions. Another obstacle relates to the usually inflexible nature of face-to-face training: participants must take the entire training course, regardless of their prior knowledge and current needs. With the growth of Internet usage worldwide, new developments have occurred in the way people gather information; as a result, information providers are increasingly using this medium to transfer knowledge to their target groups [22]. In particular, the use of e-learning modules could be an effective technique to transfer adolescent suicide prevention knowledge to gatekeepers. “E-learning,” also known as computer-based learning, online learning, distributed learning, or Web-based learning describes the use of computers to transfer knowledge to learners primarily through an intranet or the Internet [23]. This method has several advantages over traditional face-to-face training.

First, Web-based training is accessible from any location from which the gatekeeper has access to the Internet. Second, because information on the process of recognition, guidance, and referral of suicidal adolescents is presented in short separate modules, gatekeepers can customize their training. Lastly, this type of training can be composed and maintained with limited resources and as a result can be offered at a low price. Thus, gatekeepers could have easy, fast, and instant access to needed knowledge with regard to adolescent suicide prevention any time and from any location. Additionally, they can refresh their knowledge whenever needed. In 2012, a systematic review was carried out aiming to provide a first overview of existing e-learning modules on suicide prevention designed for gatekeepers, and their efficacy [24]. In that study, a Google search showed that worldwide e-learning modules were increasingly available on the topic. A literature search, however, yielded no published papers on the same topic. The results of this review highlighted the need for research, especially RCTs, on the efficacy of educational suicide prevention e-learning modules for gatekeepers [24].

In 2011, VU University in Amsterdam started a program entitled Mental Health Online (MHO), with an aim to develop adolescent suicide prevention e-learning modules for gatekeepers and to test the efficacy of these modules [25]. A total of 8 e-learning modules were developed, each capturing an important aspect of the process of recognition, guidance, and referral of suicidal adolescents (12-20 years old). The content of the modules followed the QPR model, focusing on essential knowledge and frameworks that enhance early detection, assistance, and referral of adolescents at-risk for suicide. Although the QPR Institute has also made QPR Gatekeeper Training available on the Internet, we decided not to use that version because it focuses on “suicidal people” in general, while we aimed only to address adolescent suicidality in the e-learning modules of the MHO program. Further, Web-based QPR Gatekeeper Training takes approximately 1 hour to complete; in contrast, for this study, we chose to divide the process of recognition, guidance, and referral into short modules, so that participants could customize their training based on their previous knowledge and experience. Lastly, training licenses for Web-based QPR Training become available only after paying a fee. It was expected that payment requirements would affect the willingness of gatekeepers to participate in this study.

In this paper, the results of an RCT addressing the efficacy of the MHO program are presented. Efficacy of the program was determined by measuring change in (1) actual knowledge, (2) perceived knowledge, and (3) perceived self-confidence of gatekeepers after training compared to a waitlist control group. It was expected that gatekeepers’ actual knowledge, perceived knowledge, and perceived self-confidence with regard to adolescent suicide prevention would improve after attending the MHO program compared to those in the waitlist control group. It is important to point out that the MHO program was a stand-alone program and not part of a multi-prolonged approach. To our knowledge, this is the first time that the efficacy of a Web-based adolescent suicide prevention gatekeeper training program has been investigated in an RCT.

## Methods

### Protocol

The study protocol for *Mental Health Online* was approved by the Medical Ethics Committee of VU University Medical Centre Amsterdam (registration number 2009/328), and a detailed study protocol for this RCT can be found elsewhere [26]. The first group of participants started the study in the second half of 2012, and the last group of participants finished the study in the second half of 2013.

### Design

This study was a randomized trial with a *pretest, posttest, and 3-month follow-up design* with two arms: an experimental group and a waitlist control group. The intervention was not masked. The experimental group received the intervention during the study, and the waitlist control group received the intervention after completion of the study. Participants did not receive any type of compensation for participation in this study.

### Participants

The participants of this study were Dutch-speaking gatekeepers who worked with adolescents. The inclusion criteria were the following: (1) gatekeepers 18 years of age and older, (2) who worked frequently with adolescents from 12 to 20 years of age, (3) whose profession involved responsibilities with regard to the (mental) health care of adolescents, and (4) who had access to the Internet. Although every individual who met the inclusion criteria was eligible to participate in this study, three main target groups were identified for recruitment: members of mental health care teams of schools, youth health care nurses, and (mental) health care employees.

### Intervention: MHO Program

#### Overview

**Figure 1:**
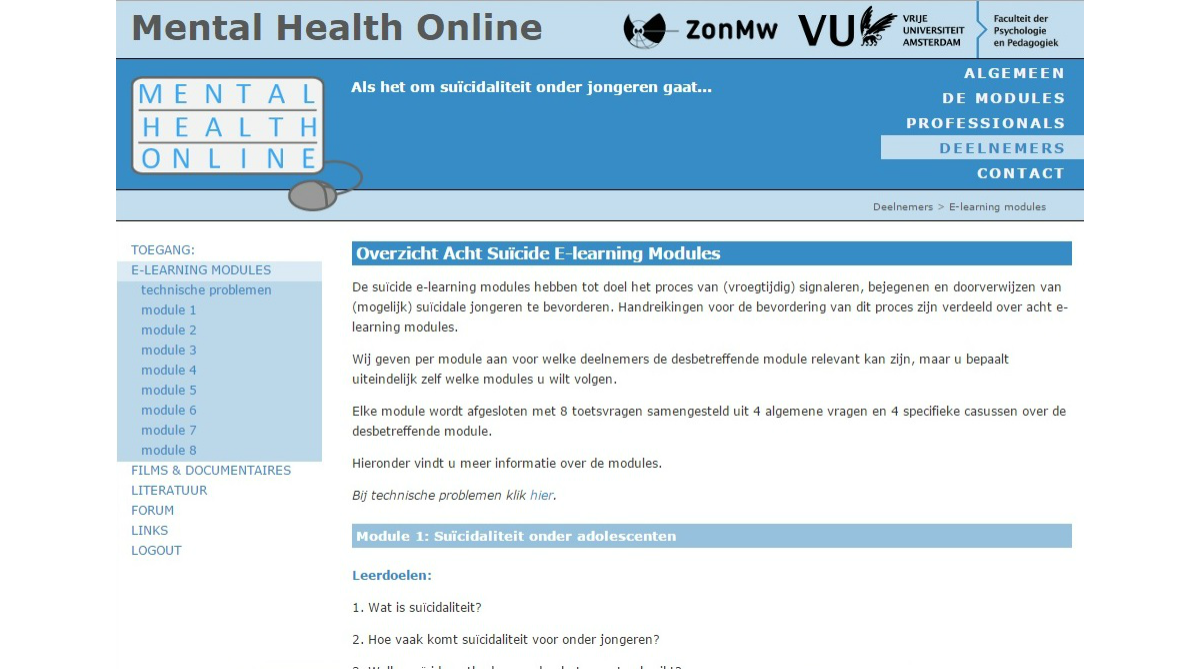
Overview of modules and additional information on the Mental Health Online website.

**Figure 2:**
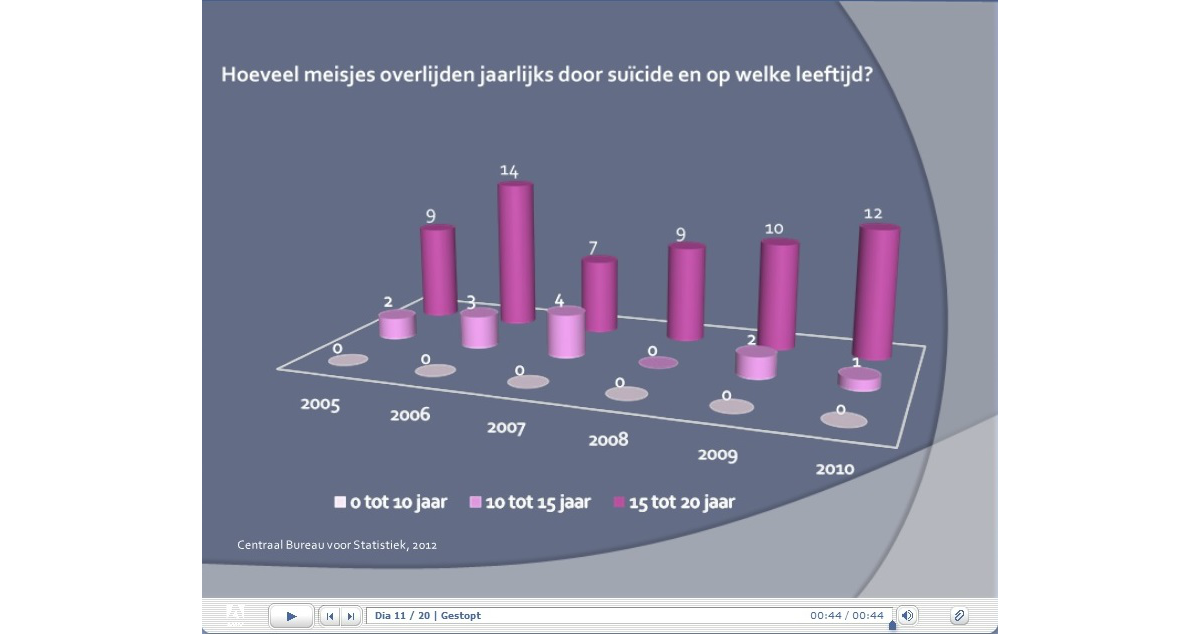
Layout of the e-learning modules of the Mental Health Online program.

The intervention in this study consisted of 8 e-learning modules alongside additional information regarding adolescent suicide prevention. The base of the modules was a PowerPoint presentation containing features such as voice-over, case descriptions, and quizzes. Both the modules and the additional information were made accessible through the website [27] for participants of this study. Figure 1 depicts a screenshot of the website (overview of e-learning modules and additional information) and Figure 2 illustrates a screenshot of one of the e-learning modules. Each of the modules of the program addressed an important aspect of the process of recognition, guidance, and referral of suicidal adolescents (12-20 years old). With an aim to allow participants to customize their training based on their previous knowledge and needs, 8 separate modules were offered. Thus, the number and order of modules were individually determined by each participant. As it was expected that the number of modules each participant followed could influence scores on the three outcome measurements of this study, a user-track system was enabled on the website. With this system, it was possible to collect data regarding how many modules each participant had completed at each assessment point.

#### E-Learning Modules

The first module was titled “Suicidality among adolescents” and gave a general introduction to the topic of adolescent suicidality, including statistics and figures. Risk factors associated with adolescent suicidality were discussed in the second module entitled “Risk factors.” The third module, “Ethnicity,” addressed the relationship between ethnicity and adolescent suicidality in the Netherlands. Warning signs associated with adolescent suicidality were presented in the fourth module entitled “Recognition of suicidality.” The fifth and sixth modules titled “Conversation with the suicidal adolescent” and “Conversation with the parents” discussed needed tools and skills when engaging in conversation with suicidal adolescents or their parents. A seventh module titled “Suicide first aid” provided practical information about how first aid should be given once an adolescent attempts suicide. The eighth and final module titled “Care and aftercare” was specifically designed for schools and offers guidelines needed to arrange the process of care and aftercare after suicide (attempt) of a student. Each module took approximately 4 to 10 min to complete.

#### Additional Information

Since the aim was to create short modules, only essential information needed to capture the purpose of the module was included. As a result, additional information (literature, documentaries, and links to other informative websites) on (adolescent) suicidality were located in a separate section of the website for those needing more material. Furthermore, a Web-based discussion board was created where participants could interact with each other, and also ask questions of experts or present cases regarding adolescent suicidality.

### Instruments

#### Overview

The MHO program was developed specifically for this study as there were no suitable instruments available. Three Web-based self-report questionnaires were developed to measure the outcomes of this study. The questionnaires were not modified from prior studies but were newly developed in collaboration with an expert in the field of suicide prevention in the Netherlands. The outcomes were (1) participants’ answers to questions tapping their actual knowledge, and their ratings of (2) perceived knowledge, and (3) perceived self-confidence with regard to adolescent suicidality and suicide prevention. The 3 questionnaires were completed by the participants at the 3 assessment points: pretest (baseline assessment, T), posttest (second assessment, T_1_) and follow-up (third assessment, T_2_). In addition, at the beginning of the follow-up assessment, participants in the experimental group were asked 2 questions about implementation of their gained knowledge. Furthermore, during the baseline assessment demographic information was gathered. Lastly, participants in the experimental group were asked to complete an evaluation questionnaire during the posttest, which aimed to assess to what extent they were satisfied with different aspects of the program and which modifications they thought could improve the program. Results of the evaluation questionnaire (including insights regarding the construction of the e-learning modules) are not presented in this paper; however, they are discussed in a separate paper (personal communication by Ghoncheh, April 16, 2015).

#### Actual Knowledge Questionnaire

The Actual Knowledge Questionnaire consisted of 6 cases each providing several characteristics (name, age, and education) of a fictional adolescent displayed in a photograph. The purpose of the photograph was to help the user visualize the adolescent and his/her situation better. Each case was followed by 2 general questions (yes/no answer), and 8 specific questions (multiple choice, 1 correct answer) each pertaining to the content of one of the e-learning modules of the MHO program. The total number of questions each participant received depended on their answers to the 2 general questions. Scores per case could vary from 0 (wrong answers to all questions) to 10 (correct answers to all questions). Two cases were presented at each assessment point: a case about a native Dutch adolescent, and an adolescent originating from an ethnic minority group in the Netherlands. Since 3 items of this questionnaire were conditional and the items were not related to each other, psychometric characteristics for this questionnaire could not be tested.

#### Perceived Knowledge Questionnaire

The Perceived Knowledge Questionnaire consisted of 9 statements to be rated on a 3-point Likert scale (0 = disagree, 1 = partially agree, 2 = agree). The first item of the questionnaire was a general statement regarding knowledge about adolescent suicide prevention (“I have sufficient knowledge about the process of recognition, guidance, and referral of suicidal youth”) [26], and the following 8 items each captured the essence of one of the e-learning modules of the MHO program. For instance, the fifth module addressed how to engage in a conversation with a suicidal adolescent and the corresponding statement was “I have sufficient knowledge to engage in a conversation with a suicidal adolescent” [26]. The scores could vary from 0 (disagreed with all statements) to 18 (agreed with all statements). During pretest, posttest, and follow-up the participants received the same questionnaire. Principal component analysis (PCA) revealed the presence of one component. The Cronbach alpha coefficient for the perceived knowledge questionnaire was .89 at pretest (experimental .89, waitlist control .90), .93 at posttest (experimental .88, waitlist control .87), and .92 at follow-up (experimental .82, waitlist control .88).

#### Perceived Self-Confidence Questionnaire

A 16-item questionnaire was developed, which consisted of statements regarding the necessary skills and attitudes when dealing with adolescent suicide prevention. The statements were rated on a 3-point Likert scale (0 = disagree, 1 = partially agree, 2 = agree) and were related to the 8 e-learning modules. “I can adequately provide first aid to a young person who has attempted suicide” and “I can make a distinction between my duties and those of a therapist” are 2 of the statements included in this questionnaire [26]. The scores could vary from 0 (disagreed with all statements) to 32 (agreed with all statements). The same questionnaire was used at each of the 3 assessment points. PCA revealed the presence of one component. The Cronbach alpha coefficient for the perceived self-confidence questionnaire was .93 at pretest (experimental .93, waitlist control .92), .95 at posttest (experimental .93, waitlist control .93) and .95 at follow-up (experimental .91, waitlist control .94).

### Recruitment

Recruitment for this study was carried out in the second half of 2012 and lasted approximately 3 months. A broad and stepwise recruitment strategy was used. First, the domain name [27] was registered and information regarding the study was posted on the website. Second, almost all education partnerships in the Netherlands were contacted by email and asked to distribute the email to their mailing list. In addition, those interested were given the opportunity to invite the main researcher of this study for an on-site presentation. Third, several information websites that are followed by gatekeepers were asked to place a summary of the research and a link to MHO on their website. Fourth, the main researcher attended seminars and conferences also attended by potential participants and handed out flyers. Fifth, VU University Amsterdam released a press release about the study that was distributed through several newsletters, and lead to 2 interviews with national newspapers. Lastly, Twitter and Facebook accounts were created for this study. Promotional materials regarding the study and up-to-date information about the study were shared with followers on both accounts.

### Procedure

As this was a Web-based study, every aspect took place on the Internet, including communication and data collection, which was done by the main researcher. All participants were required to register by sending an email and including their name, position, affiliation, and email address. The baseline assessment was sent to participants by email; after completion of this assessment, participants were randomized to either the experimental or control group. One week after completing the baseline assessment, participants assigned to the experimental group received a personal username and password, along with a guide to the website. The login information gave each participant access to the website for 14 days.

Four weeks after completing the baseline assessment, the link to the second assessment was sent to the participants by email. After finishing the second assessment, those in the experimental group regained access to the website until 1 week prior to receiving the third assessment. The link to the third and final assessment was sent to the participants 12 weeks after finishing the second assessment. After completing the third assessment, participants in the waitlist control group were given access to the website through an email containing a personal username and password. At the same time, those in the experimental group also received an email in which they were notified that they had regained access to the website, in case they wanted to refresh their knowledge or use the additional information.

Participation was monitored by the main researcher and participants received reminders or were contacted if necessary.

### Data Analyses

All analyses were carried out on the intention-to-treat sample. Hierarchical linear modeling (HLM) was conducted in MLwiN version 2.28 to determine whether differences between the two groups existed in actual knowledge, perceived knowledge, and self-confidence after the experimental group received the intervention. MLwiN integrates data from participants missing one or more measurements, or 1 or more questionnaires into the analysis. A 2-level HLM was conducted for each outcome measure (perceived knowledge, perceived self-confidence, and actual knowledge) where the outcome measures (level 1) were nested within gatekeepers (level 2). In order to determine the intervention effect, 2 separate models were tested for each of the 3 outcome measures. The first model explored the overall effect of the intervention across time correcting for the baseline assessment. The second model explored the effects of the intervention at posttest and follow-up by adding the interaction term (*group* × *time*) to the previous model. Other analyses were conducted using IBM SPSS Statistics version 21.

## Results

### Response Rates

**Figure 3:**
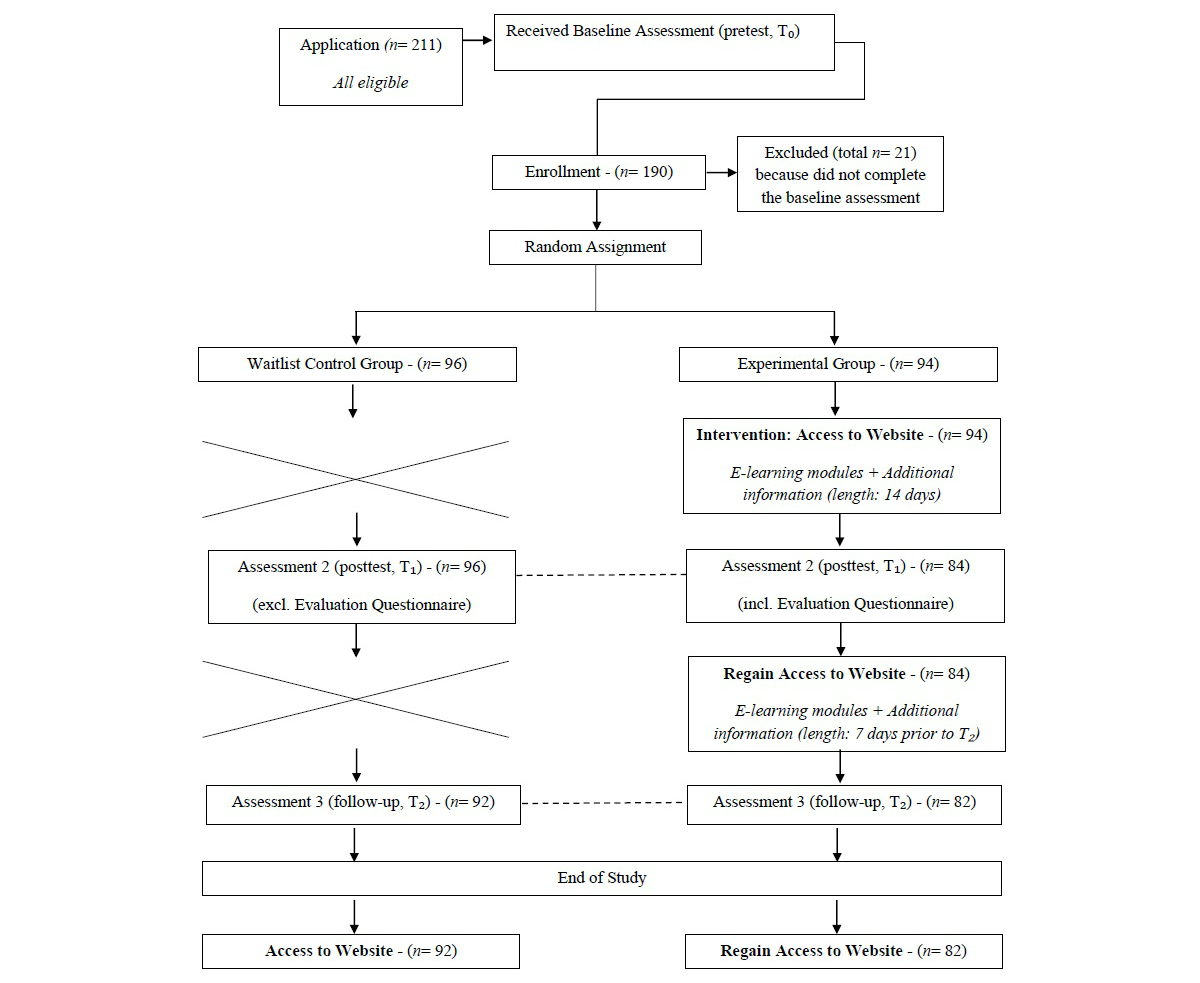
Flow of participants through each stage of the study.

A total of 211 gatekeepers registered for the study, of which 190 completed the baseline assessment and were enrolled. The enrolled participants were randomized to either the experimental group (n=94) or the waitlist control group (n=96). In the experimental group, 4 participants did not follow the e-learning modules and subsequently did not receive the second assessment. The remaining 90 participants received the second assessment and 84 completed the second assessment (response rate 89.4%, 84/94). All participants in the waitlist control group completed the second assessment (response rate 100%). The third assessment was completed by 82 participants in the experimental group (response rate 87.2%, 82/94) and 92 participants in the waitlist control group (response rate 95.8%, 92/96). Figure 3 illustrates the flow of participants through each stage of the study.

The 16 participants who dropped out of the study were contacted by the main researcher. The following reasons were given by the participants for not completing the study: lack of time (n=7), family circumstances (n=2), unable to open the questionnaire at work and lack of time to fill out the questionnaire at home (n=2), pregnancy leave (n=1), absence due to vacation (n=1), and objection regarding the nature of testing (n=1). The remaining 2 participants did not respond.

No differences were found between groups with regard to mean scores of participants who completed the study and those who dropped out: actual knowledge at pretest (*t*_188_ = 1.271, *P*=.21, two-tailed), actual knowledge at posttest (*t*_180_ = 1.709, *P*=.09, two-tailed), perceived knowledge at pretest (*t*_188_ = -0.200, *P*=.84), perceived knowledge at posttest (*t*_182_ = 1.107, *P*=.27, two-tailed), perceived self-confidence at pretest (*t*_188_ = 0.269, *P*=.79, two-tailed), and perceived self-confidence at posttest (*t*_181_ = -0.168, *P*=.87, two-tailed).

### Descriptive Analysis

Gatekeepers in this study were 21 to 62 years of age (mean 43.55, SD 10.96), the majority were female (81.6%, 155/190) and had a higher vocational (55.8%, 106/190) or university (38.4%, 73/190) degree. The majority (67.9%, 129/190) of the gatekeepers worked within a school setting (such as mentors, counselors, teachers, and social workers) while the rest worked in a (mental) health care related setting or institute (such as psychologists, behavioral scientists, youth health care nurses, and psychiatrists). The participants of this study had 0 to 30 years of experience in their current job (mean 8.28, SD 7.16). Moreover, 78.9% (150/190) of the participants reported knowing at least one adolescent who performed a nonfatal suicide attempt, and 39.5% (75/190) of gatekeepers reported knowing at least one adolescent who died due to suicide. All participants were from the Netherlands, except one gatekeeper who lived in Belgium. No differences were found between the experimental group and waitlist control group in terms of demographics.

**Figure 4:**
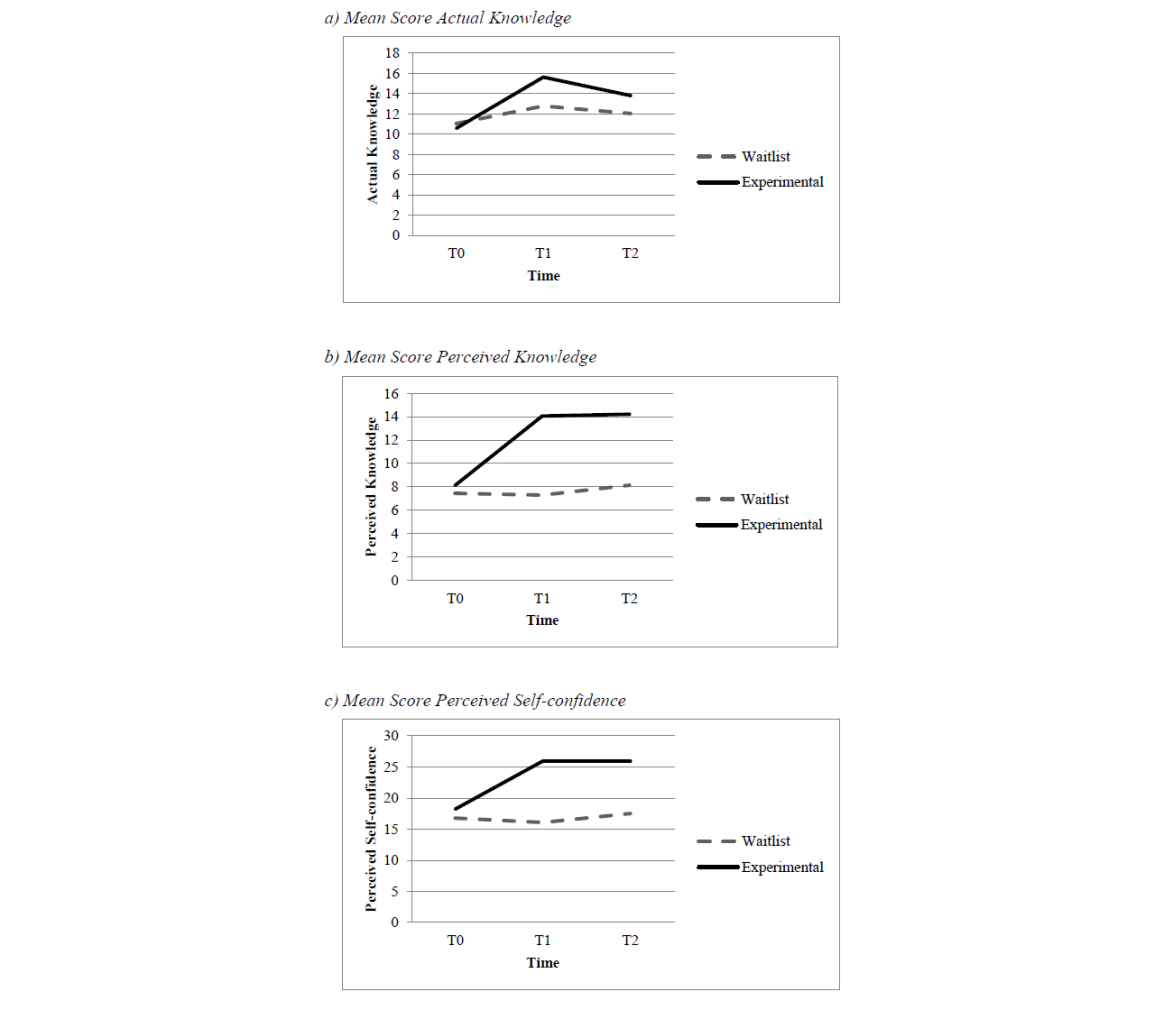
Mean scores of the groups on the 3 questionnaires at T_0_, T_1_, and T_2_.

Table 1 shows the mean scores and standard deviations of both groups on actual knowledge, perceived knowledge, and perceived self-confidence at baseline, posttest, and follow-up. The groups’ mean scores at the 3 assessment points are also illustrated in Figure 4. At baseline, no significant differences were found between the waitlist control group and experimental group for actual knowledge (*t*_188_ = 1.106, *P*=.27, two-tailed), perceived knowledge (*t*_188_ = -1.042, *P*=.30, two-tailed), and perceived self-confidence (*t*_188_ = -1.301, *P*=.20, two-tailed).

**Table 1:** Mean scores for actual knowledge, perceived knowledge, and perceived self-confidence over time.

	Baseline (T)		Posttest (T_1_)		Follow-up (T_2_)	
Questionnaire	Waitlist Control (n=96) mean (SD)	Experimental (n=94) mean (SD)	Waitlist Control (n=96) mean (SD)	Experimental (n=88) mean (SD)	Waitlist Control (n=92) mean (SD)	Experimental (n=82) mean (SD)
AK^a^	11.05 (3.07)	10.59 (2.74)	12.79 (2.30)	15.63 (2.97)	12.05 (3.30)	13.82 (3.00)
PK^b^	7.45 (4.44)	8.13 (4.55)	7.30 (3.99)	14.07 (3.66)	8.14 (4.02)	14.22 (2.98)
PS^c^	16.78 (7.44)	18.21 (7.73)	16.08 (7.29)	25.94 (5.81)	17.52 (7.34)	25.93 (5.34)

^a^AK: actual knowledge

^b^PK: perceived knowledge

^c^PS: perceived self-confidence

### Outcome Measures Across Time by Condition

As shown in Table 2, the overall effect of the intervention was highly significant across time and resulted in large overall effect sizes (ES) for actual knowledge (ES = 0.76), perceived knowledge (ES = 1.20), and perceived self-confidence (ES = 1.02). This indicates, first, that the MHO program had a large positive effect on actual knowledge, perceived knowledge, and perceived self-confidence of the participants completing the program compared to those in the waitlist control group, and, second, that the effects were sustainable as they remained significant at 3-month follow-up. Further analyses showed that the intervention effect was strongest at posttest compared to follow-up for actual knowledge (ES = 0.94), perceived knowledge (ES = 1.30), and perceived self-confidence (ES = 1.12), and that the effects remained large for perceived knowledge (ES = 1.09), and perceived self-confidence (ES = 0.90) after 3 months. For actual knowledge, a medium effect size (ES = 0.57) was found at follow-up, indicating a decrease in the actual knowledge of the participants over 3 months.

**Table 2:** Training impact.

Variable	B	Overall Effect 95% CI	ES^a^	B	Effect at Posttest 95% CI	ES	B	Effect at Follow-up 95% CI	ES
Actual Knowledge	2.415	(1.76 - 3.07)	0.76	2.995	(2.19 - 3.80)	0.94	1.828	(1.00 - 2.65)	0.57
Perceived Knowledge	5.883	(5.12 - 6.65)	1.20	6.363	(5.52 - 7.21)	1.30	5.359	(4.92 - 6.22)	1.09
Perceived Self-Confidence	8.112	(6.82 - 9.41)	1.02	8.942	(7.49 - 10.39)	1.12	7.216	(5.74 - 8.69)	0.90

^a^Effect size (ES) is the regression coefficient divided by the total standard deviation. All models were significant at *P*<.001.

Of the 84 participants in the experimental group who finished the second assessment, 71 (85%) completed all 8 e-learning modules of the MHO program. For this reason, further analyses exploring whether the number of e-learning modules a participant completed had an effect on actual knowledge, perceived knowledge, and perceived self-confidence were not conducted.

### Application of Gained Knowledge

At 3-month follow-up, 45%, 37/82) of gatekeepers from the experimental group reported that they had applied the knowledge gained over the past 3 months. According to the 36 respondents who elaborated on their answer, this application of knowledge was done in the following way: recognition of and/or engaging in conversation about suicidality (n=25), all the steps from recognition to referral (n=5), advising and sharing knowledge with other gatekeepers (n=3), awareness (n=2), and other (n=1).

## Discussion

### Principal Findings

This RCT investigated the efficacy of a Web-based adolescent suicide prevention gatekeeper training problem (MHO), consisting of 8 e-learning modules and additional information. The results of this study show that the actual knowledge, perceived knowledge, and perceived self-confidence of gatekeepers who enrolled in the MHO program improved significantly compared to gatekeepers who did not have access to the program, and that the effects found immediately after the training remained significant at 3-month follow-up. Moreover, almost half of the participants that accessed the training program reported using the knowledge gained at least once during the 3-month follow-up.

Our findings are in accordance with previous studies that investigated the efficacy of QPR Gatekeeper Training delivered face-to-face to gatekeepers working with adolescents [11,19-21]. These studies also found gains in (perceived) knowledge and perceived self-confidence of gatekeepers attending the training. To our knowledge, Wyman and colleagues (2008) have conducted the only RCT assessing the impact of QPR Gatekeeper Training in gatekeepers working with adolescents. In their study, a large effect size was found for perceived knowledge (ES = 1.32) and perceived efficacy (ES = 1.22), and a medium effect size (ES = 0.41) was found for QPR knowledge at 1-year follow-up [19]. Similar to the study of Wyman et al, we found a large effect size for training on perceived knowledge and perceived self-confidence, and a medium effect size for actual knowledge at (3-month) follow-up. Since we measured the participants three times, we were also able to estimate the effect sizes across time and immediately after finishing the training. For both of these analyses, we found large effect sizes for the three outcome measurements.

Although this study suggests that a Web-based training program has similar effects as face-to-face training with respect to training gatekeepers in adolescent suicide prevention, the current study did not compare Web-based training to face-to-face training, and to the best of our knowledge, other researchers have not yet compared the two formats. As a result, it remains unclear whether Web-based adolescent suicide prevention training for gatekeepers is actually as effective as face-to-face training. In-person interaction with the trainer and other participants and the opportunity to practice gained knowledge during role-play are probably the most important advantages of face-to-face training compared to distance learning. However, it remains questionable whether these elements actually contribute to additional increases in knowledge, self-confidence, and skills of gatekeepers, as the only way to implement and practice gained knowledge for the participant is to interact with a suicidal adolescent, which is similar for Web-based learning. As long as we cannot test the effects in real-life interactions between gatekeepers and suicidal adolescents, it remains unclear to what extent the outcome measurements really have increased as a result of training (Web-based or face-to-face). The fact that almost half of the participants in this study stated that they had implemented the knowledge gained during the 3-month follow-up suggests that it indeed led to increased self-confidence and implementation of the required steps, the latter of which could also indicate skill improvement.

Future research is needed to replicate the findings of this study and to determine which features enhance learning outcomes. As noted, we also asked participants to evaluate the MHO program, to understand better the program improvements that gatekeepers need. The results of the evaluation are discussed in a separate paper (personal communication by Ghoncheh, April 16, 2015). Furthermore, future research should also investigate to what extent Web-based learning can replace or supplement existing traditional educational strategies in suicide prevention. Even if results of future research favor traditional methods compared to Web-based training, for example regarding acquired skills, the results of this study showed that Web-based training is effective in knowledge gain and self-confidence enhancement. Thus, based on the findings of this study, we recommend that evidence-based Web-based adolescent suicide prevention training programs should be offered as base training to gatekeepers. Due to the accessibility and flexibility of Web-based training, gatekeepers—as many as possible—will become familiar with the necessary steps in adolescent suicide prevention. This will likely result in detecting more adolescents in need and referring them to professionals who can assist them. Thereafter, those in need of more in-depth information and personal interaction or practice opportunities can attend face-to-face training. Subsequently, this could be beneficial to face-to-face training, as a more homogenous group of gatekeepers would attend, and custom content could be created for those looking for advanced material on adolescent suicide prevention.

The findings of this study have potential implications for education on prevention of other mental health issues. Although this study focused on adolescent suicide prevention, its results show that Web-based training is a promising tool for gatekeepers’ education and that the findings are probably generalizable to other topics. Gatekeepers can be easily educated on various and highly important topics such as adult suicide, depression, and eating disorders, as well as child/adolescent behavioral, emotional, and developmental problems and disorders. For example, Dutch gatekeepers, especially those working in schools, may benefit from the advantages of Web-based training, primarily because the government has assigned them with prevention and intervention responsibilities concerning the (mental) health care of their students [28]. As such, adolescents at-risk can be detected early and referred for help.

This study has several strengths. It is innovative in being the first RCT investigating the efficacy of educational suicide prevention e-learning modules for gatekeepers working with youth. Being an RCT, it yielded reliable findings obtained in a design with sufficient statistical power. However, the study findings should be interpreted in light of several limitations. A possible, yet inevitable, limitation of this study is that no standardized instruments were available to test the outcome measurements. Nevertheless, the perceived knowledge and perceived self-confidence questionnaires had high reliability across the three measurements and PCA revealed the presence of one component for both questionnaires. Unfortunately, psychometric characteristics of the actual knowledge questionnaire could not be tested as the item content was based on specific cases and several questions were conditional. Second, although 45% of the participants mentioned that they had put gained knowledge from the modules into practice during the 3-month follow-up, due to privacy reasons, it was not possible to monitor the gatekeepers who participated in this study or to obtain actual information on referrals they made. As a result, we could not measure changes in actual suicide prevention skills and performance. Future research should determine whether distance learning actually improves the behaviors of gatekeepers necessary for preventive activities and eventually leads to greater detection of suicidal adolescents, and correct referrals. Third, although we included a 3-month follow-up, maintenance of the intervention effects across a longer period was not ascertained.

### Conclusion

Despite its limitations, this study is of value for gaining insight into the potential of e-education for professionals involved in the field of prevention of undesirable outcomes. It is the first study that tested the efficacy of adolescent suicide prevention e-learning modules targeting gatekeepers in an RCT. The findings are promising and provide evidence that the use of Web-based resources, such as e-learning modules, could be an effective strategy in the improvement of gatekeepers’ actual knowledge, perceived knowledge, and perceived self-confidence in adolescent suicide prevention. Future research is needed to support the findings of this study.

## Abbreviations

AK: actual knowledge
ASIST: Applied Suicide Intervention Skills Training
ES: effect size
HML: hierarchical linear modeling
MHO: Mental Health Online
PK: perceived knowledge
PS: perceived self-confidence
QPR: Question, Persuade, Refer
RCT: randomized controlled trials
SOS: Sources of Strength
WHO: World Health Organization

## Appendix

Multimedia Appendix 1 Consort eHealth checklist.
